# Trifurcation of the right common carotid artery

**DOI:** 10.4103/0970-0358.41121

**Published:** 2008

**Authors:** R. Chitra

**Affiliations:** Department of Anatomy, Siddhartha Medical College, Vijayawada, Krishna District, Andhra Pradesh, India

**Keywords:** Ascending pharyngeal artery, common carotid artery, external carotid artery, lateral position, trifurcation

## Abstract

Variations in the position of the bifurcation of the common carotid artery and the origin or branching pattern of the external carotid artery are well known and documented. Here, we report the trifurcation of the right common carotid artery in a male cadaver aged about 55 years. The right common carotid artery was found to divide into the external and internal carotids and the occipital artery. High division of bilateral common carotid arteries and a lateral position of the right external carotid artery at its origin were also observed in the same cadaver. There were two ascending pharyngeal arteries on the right side - one from the occipital artery and another from the internal carotid artery. The intraarterial approach is one of the most important routes for the administration of anticancer drugs for head and neck cancers. A profound knowledge of the anatomical characteristics and variations of the carotid artery such as its branching pattern and its position is essential to avoid complications with catheter insertion.

## INTRODUCTION

The right common carotid artery originates in the neck from the brachiocephalic trunk while the left arises from the aortic arch in the thoracic region. The cervical portions of the common carotids resemble each other very closely. The common carotid artery is contained in a sheath known as the carotid sheath, which is derived from the deep cervical fascia and also encloses the internal jugular vein and vagus nerve, the vein lying lateral to the artery and the nerve between the artery and vein on a plane posterior to both. At approximately the level of the fourth cervical vertebra, the common carotid artery bifurcates into an internal carotid artery and an external carotid artery in the carotid triangle. The external carotid artery lies anteromedial to the internal carotid artery at its origin but becomes anterior and lateral as it ascends. In the neck, the external carotid artery gives off six branches: superior thyroid, lingual, facial and occipital, ascending pharyngeal and posterior auricular arteries. Variations of the common carotid artery include the rare absence of the common carotid artery,[1,2] the high or low bifurcation of the common carotid artery and also the abnormal branches of the common carotid artery such as the superior thyroid artery or even the thyrolingual trunk.[3]

## MATERIALS AND METHODS

The carotid system of arteries were observed for variations in 25 cadavers for the period of 3 years from 2004-2007, in routine educational dissection for undergraduate students. In the academic year of 2006-2007 in our department, this variation of right common carotid was observed in a male cadaver aged about 55 years. The right common carotid artery divided into the external and internal carotids and the occipital artery. High termination of both common carotid arteries and the lateral position of the right external carotid artery at the origin were also observed in the same specimen. The branching pattern was also different in the right external carotid artery.

## RESULTS

**Figure 1:**
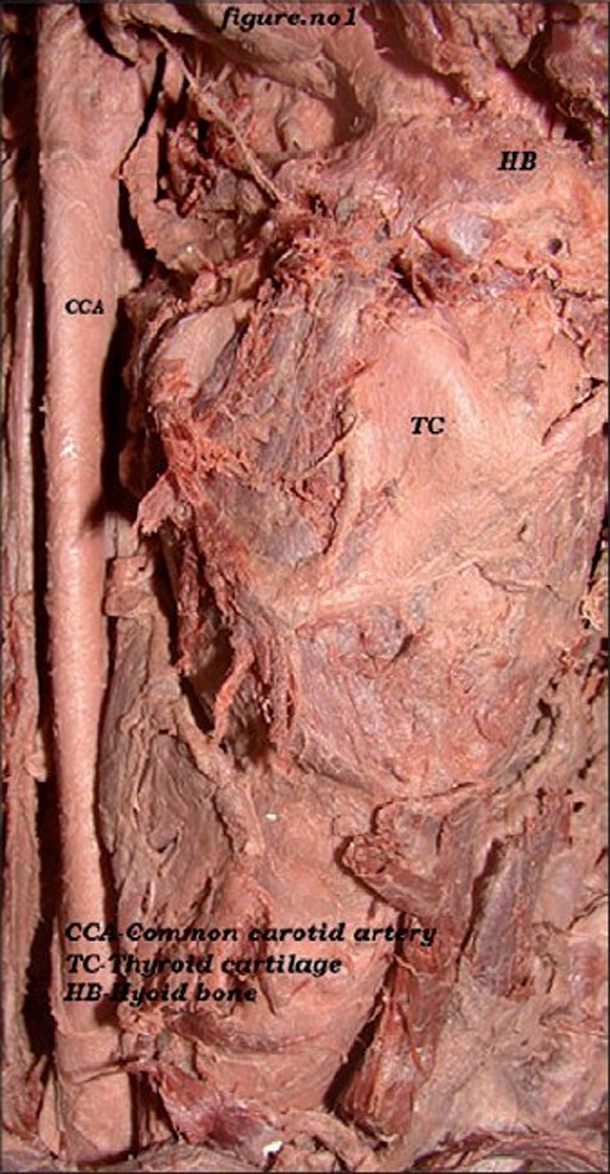
High bifurcation of the right common carotid artery

**Figure 2:**
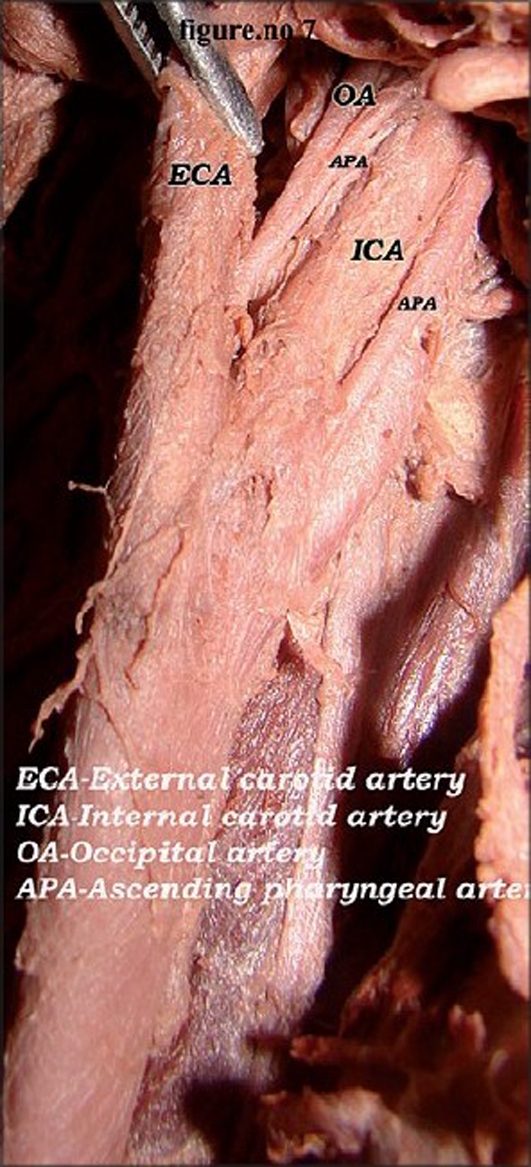
Lateral position of the right external carotid artery

**Figure 3:**
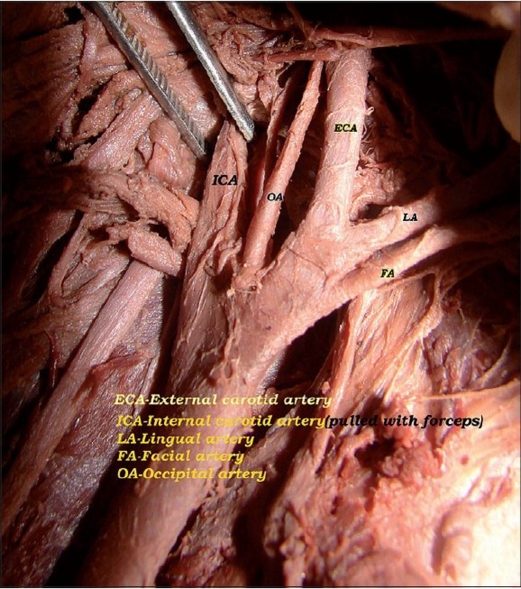
Branches of the right external carotid artery

**Figure 4:**
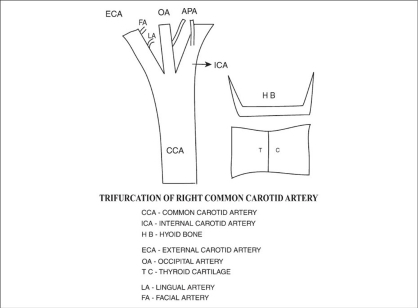
Line diagram of the trifurcation of the right common carotid artery

In the male cadaver, on the right side, the common carotid artery divided at the higher level coinciding with the level of the tip of the hyoid bone. The length of the right common carotid artery was 10.5 cm [Figure 1]. At its division, the external carotid was anterolateral and the internal carotid artery was posteromedial [Figure 2]. The occipital artery arose at the carotid bifurcation characterizing it as a trifurcation of the right common carotid artery. The ascending pharyngeal artery was the branch of the occipital artery [Figure 2]. Another ascending pharyngeal artery arose from the right internal carotid artery [Figure 2]. The superior thyroid artery arose from the common carotid artery. Facial and lingual arteries arose from the external carotid artery as the first and second branches [Figure 3]. The line diagram of the trifurcation of the right common carotid artery is shown in Figure 4. The remaining branches of the right external carotid artery were normal in origin. On the left side, the common carotid artery bifurcated at the same higher level but the positions of the external and internal carotid arteries were normal. The superior thyroid artery rose from the left common carotid artery. The other branches of the left external carotid artery were normal in origin.

## DISCUSSION

The origin of the occipital artery from the carotid bifurcation has been reported by Quain,[4] Livini,[5] Gurburz *et al.*[6] In a large study Adachi *et al.*[7] studied 298 subjects and described only two cases of the left occipital artery branching off the carotid bifurcation, characterizing a trifurcation of the carotid tripod. Marques *et al.*[8] observed the occipital artery arising at the carotid bifurcation in two cases out of 110 cases which closely resembled those described by Gluncic *et al.*[9]

The position of the carotid bifurcation reflects the degree of embryological migration of the external carotid artery and is variable. Huber[10] reports the bifurcation at C4 to C5 in 48% and at C3 to C4 in 34% of 658 bifurcations.

The first report of a lateral position of the external carotid artery was that of Hyrtl[11] in 1841. In about 80% of patients, the internal carotid artery arises posterior or posterolateral to the external carotid artery. Teal[12] found that in 4% of patients, the internal carotid artery is medial and in 8%, the internal carotid artery is posteromedial.

Lasjaunias *et al.*[13] have described in detail the phylogenetic and embryological basis of the common origin of the ascending pharyngeal and occipital arteries as well as their origin from the cervical segment of the internal carotid artery. They describe a pharyngo-occipital system located at the craniocervical junction consisting of the ascending pharyngeal and occipital arteries as they together supply the three cervical somites C1, C2, C3 and the third branchial arch. The pharyngo-occipital system explains the variability in the origin of the ascending pharyngeal and occipital arteries as well as their common origin.

Furthermore, Lasjaunias *et al.*[13] believe that the cervical segment of the internal carotid and ascending pharyngeal arteries developmentally share a relationship with the third aortic arch. The cervical segment of the internal carotid artery is derived from the third aortic arch and the ascending pharyngeal artery may represent the dorsal vestige of the third aortic arch. This may explain the origin of the ascending pharyngeal artery from the cervical segment of the internal carotid artery.

Carotid endarterectomy is the main treatment for atherosclerotic plaques of the cervical internal carotid artery. The branches of the external carotid artery are the key landmarks for adequate exposure and appropriate placement of cross-clamps on the carotid arteries. It is necessary to understand the surgical anatomy of the carotid arteries to carry out successful removal of plaques and minimize postoperative complications in a bloodless surgical field. Transcatheter embolization procedures in the external carotid artery are largely used on hypervascular tumors, epistaxis and trauma

## CONCLUSION

The patterns of variability in the branches of the carotid artery are of paramount importance not only in clinical practice but also in theoretical considerations.[14] Among the arterial branches in the human body, the carotid bifurcation is particularly important because the internal carotid artery supplies blood to the brain. Lack of experience regarding the possible variations could lead to fatal errors if one blood vessel is mistaken for another.[15] A profound knowledge of the anatomical characteristics and variations of the carotid artery such as its branching pattern and its position is essential to avoid complications with catheter insertion of carotid arteries in various procedures. This important variation could lead to severe complications when radiographic evaluation or surgical proceedings are done in the neck without any prior knowledge.
